# Teaching therapy decision-making to medical students: a prospective mixed-methods evaluation of a curricular innovation

**DOI:** 10.1186/s12909-024-06421-y

**Published:** 2024-12-26

**Authors:** Diego Garcia-Huidobro, Joaquín Fernandez, Pilar Espinosa, Nicole Lustig, Ignacio Perez, Luz M. Letelier

**Affiliations:** 1 Pontificia Universidad Catolica de Chile, Vicuña Mackenna, Macul, Santiago Chile; 2 University of Minnesota, Minneapolis, MN 55455 USA

**Keywords:** Therapeutic reasoning, Therapy decision-making, Medical school, Medical education, Medical students, Medicine

## Abstract

**Background:**

Therapy decision-making (TDM) is an essential medical skill. However, teaching therapeutic reasoning poses significant challenges. We present a comprehensive TDM course for medical students and report on student satisfaction with the educational strategies, their perceived importance of various TDM domains, and their self-efficacy in incorporating these elements into clinical decisions.

**Methods:**

Three student cohorts participated in a 16-week TDM course, which included self-instruction modules, application assignments, faculty symposia, and application seminars as educational strategies. The course focused on TDM and emphasized how factors such as the patient’s diagnosis, needs and preferences, treatment options, physicians’ viewpoints, the patient-physician relationship, and contexts of medical practice impact TDM. After the course, students completed a before-and-after survey assessing their satisfaction with the educational strategies, their perceived importance of ten TDM domains, and their ability to incorporate these domains into patient management. Scores ranged from 1 to 10. Students from the first two cohorts completed a 1- and 2-year follow-ups.

**Results:**

A total of 387 students completed the course. All educational strategies were well-received, with self-instruction modules and faculty symposia yielding the highest satisfaction rates (94.8% and 88.6% respectively). Before-and-after evaluations indicated that students` perceived importance of the TDM domains increased from an average of 8.0 ± 2.4 at baseline to 9.9 ± 1.0 after the course. Additionally, their perceived ability to integrate TDM domains into practice rose from an average of 5.2 ± 3.2 to 9.4 ± 1.5 by the end of the course. Follow-up results showed a decrease in these outcomes over time.

**Conclusion:**

This course serves as a successful model for systematically teaching TDM to medical students.

**Supplementary Information:**

The online version contains supplementary material available at 10.1186/s12909-024-06421-y.

## Introduction

### Practice Points

- Therapy decision-making is a critical skill for medical practice. However, many medical graduates are not adequately prepared to prescribe treatments safely and independently.
- We present a comprehensive course on therapy decision-making for medical students. The course focuses on ‘how’ therapy decisions are made and incorporates self-instruction modules, application assignments, faculty symposia, and application seminars as learning strategies.
- After completing the course, participants reported an increased perceived importance of various aspects for therapy decision-making and an improved ability to incorporate these elements into their own therapeutic decisions.
- Medical schools should formally integrate therapeutic reasoning courses into their curricula. These courses should cover the reasoning behind therapeutic decisions, explore the diverse treatment options available in medicine, include content on key domains essential for therapy decisions, and encourage the sharing of personal experiences between students and experienced physicians.

Therapeutic reasoning or therapy decision-making (TDM) is a critical medical skill as it goes beyond diagnostic reasoning to involve the identification and application of the best therapeutic options for patients, considering a complex web of factors. TDM involves not only clinical expertise and treatment availability but also incorporates patient preferences, the healthcare setting, regulatory guidelines, and social context [1]. The multiple aspects of therapeutic reasoning make it a difficult skill to master, [2] and inadequate prescribing remains a significant issue, with approximately 18% of prescriptions considered suboptimal, [3] affecting health outcomes, healthcare costs, and patient satisfaction [4, 5]. For this reason, medical schools recognize therapeutic reasoning as a significant need in medical education [6–8].

In contrast to diagnostic reasoning, which has received substantial attention in medical education, [9] TDM training remains underexplored, with few structured strategies available [10, 11]. Most published TDM training strategies involve case studies, [12] simulation (e.g., role playing), [13] or shadowing experienced providers [14]. The current manuscript presents a novel TDM course for medical students and reports change in students’ perception of importance of different domains for therapy decisions and their self-efficacy to incorporate these elements in their clinical management.

## Educational innovation

The Therapy Decision-Making (TDM) course is part of the clinical phase of medical training at the Pontificia Universidad Catolica de Chile (PUC) Medical School, specifically, in the 9th semester when students engage in clinical rotations. A brief description of the School of Medicine and its undergraduate curriculum is presented in Supplement 1. This 16-week course was designed as part of a Medical School curricular transformation, under which students provided informed consent to participate in evaluations of educational innovations. This curricular change allowed for greater integration of clinical knowledge and skills, and this course became the second in a four-part clinical integrative course series. In addition to this course, 4th year medical students participate in clinical rotations during the mornings and attend lectures in clinical medicine in the afternoons.

The course is designed to help students integrate various aspects of therapeutic decision-making not typically covered in traditional courses. It aims to teach students ‘how’ to make treatment decisions by considering patient and environmental factors, disease characteristics, available treatments, and personal provider preferences. Students learn to select a wide array of therapies: education, counseling, nutrition, physical therapy, pharmacotherapy, surgery, psychotherapy, and alternative therapies. A detailed course overview, following the Template for Intervention Description and Replication (TIDieR [15]), is presented in Table 1.

**Table 1:** Course description using the template for intervention description and replication (TIDieR)

TIDieR Domain
Brief Name:
1. Therapy Decision Making Course for Medical Students
Why:
2. Therapy decision-making (TDM) is an essential medical skill. However, teaching therapeutic reasoning is challenging
What:
3. Materials: 12 Self-instruction modules with specific contents outlined in Table 2; 12 application videos; 4 60-min faculty symposia; and 4 90-min application seminars
4. Procedures: On a weekly basis, students read the materials on the self-instruction modules and apply their contents to patients they had seen during their clinical rotations. Every 3 weeks, students have a symposium with physicians from different specialties to share their therapy decision making experiences. The course instructor interviews them leaving opportunities for students to share their thoughts and reactions. After these symposia, students meet with preceptors to integrate the contents studied in their self-instruction modules. In these seminars, students follow a clinical case study and make therapy decisions discussing the contents previously reviewed
Who provided:
5. Self-instruction modules and application videos are individual activities. In the faculty symposia a myriad of faculty are interviewed by the course’s instructor, including internal medicine, palliative care, nutrition and diabetes, pediatrics, psychiatry, digestive surgery, orthopedic surgery, intensive care, and emergency medicine physicians. Faculty from different medical specialties, including endocrinology, family medicine, hematology, internal medicine, oncology, otolaryngology, rheumatology, guide application seminars. Students keep the same preceptor during all application seminars
How:
6. Self-instruction modules are posted online on a weekly basis. These modules use problem-based learning. Each module has two or three clinical vignettes that question the student´s knowledge about the module´s contents. Then, the week´s content (summarized in Table 2) is presented. Finally, the questions asked at the beginning of the module are answered, applying the module´s content to the clinical vignettes. In addition, students record weekly application videos that allow them integrating the course´s contents to their clinical experiences. Videos have a maximum duration of 10 min and have to be turned in electronically on the course´s portal. Faculty symposia are held at a large auditorium and all students are invited to attend. The course´s instructor has an interview guide that initiates the conversation. Guest faculty are invited to introduce the types of therapy decisions that they routinely face in their clinical practice, and then reflect on aspects affecting their decision-making processes. Once students begin asking questions and sharing reflections, the discussion is continued from there, focusing on the application of the course contents. At the end of each symposium, guest faculty were invited to give recommendations for comprehensive therapy decision-making. Throughout the session, the course´s instructor highlights the application of the course´s contents by the guest faculty. Application seminars are held in groups of 10–13 students. Students follow a clinical case with therapy questions. Students are asked to share their previous weeks’ learning and apply it to the case. Preceptors have a guide for the session with answers to the discussion questions. In addition, they are invited to share their personal experiences during the seminar focusing on how they apply the course´s contents in their daily clinical practice
Where:
7. To execute the course, we use the university´s online portal with capacity to store course materials and allow uploading the weekly application videos, a large auditorium for faculty symposia, and about 10 small rooms for application seminars
When and How Much:
8. The course is implemented annually during the first semester of the 4th year of Medical School (that has a total of 6 years). The course lasts 16 weeks and has a dedication of 5 h per week
Tailoring and Modifications:
9. Depending on the academic calendar, the order of the self-instruction modules is organized. In addition, after reviewing the student´s evaluation the course is adjusted according to the feedback received. For example, the clinical vignettes of the application seminars have been changed to specific diseases that are known for most students, so the discussion is focused on “how” a treatment is chosen rather than “what” treatment options exist and could be used. Recently, we have included gender perspectives throughout the course. In the application seminars, we have changed the language used, we have had a similar proportion of male and female vignettes. In addition, we have aimed to have a similar proportion of male and female guest faculty during faculty symposia and application seminar preceptors
How well planned and actually implemented:
10. All the courses are very organized, and most students receive a very similar intervention. The self-instruction modules are structured learning materials, and application videos have specific guidelines for students to reflect and apply the course materials. Faculty symposia are initially guided by an interview guide, but follow-up questions are unstructured. Application seminars have a common structure following a clinical case, but each preceptor could add personal reflections or adjust the application of contents according to their medical specialty or clinical experience

Developed by DGH and LML, the course content reflects over 20 years of clinical experience across inpatient, emergency, and outpatient settings. Drawing from their reflections on therapeutic reasoning and existing TDM research, [1] the developers presented an initial proposal to the PUC’s Undergraduate Medical Education curriculum committee, incorporating their feedback into the course. The course emphasizes context-learning, [16] integrating theoretical and clinical knowledge in real-time rather than sequentially. The course dedicates 5 h per week to cover topics, sessions, and content as shown in Table 2.

**Table 2:** Course’s topics and contents

Topic	Number of sessions	Contents
Medical error and treatment uncertainty	1	• Medical error • Treatment uncertainty
Medical legislation	1	• Impact of legislation in TDM • Specific laws impacting TDM in Chile
Evidence-based medicine	2	• Impact of scientific evidence in TDM • How to evaluate the quality of systematic reviews • How to evaluate the quality of clinical practice guidelines
Treatment costs	1	• Impact of costs of treatments and cost-effectiveness in TDM • Costs in health interventions • Measures to evaluate the impact of costs of medical decisions
Ethics	1	• Ethics in TDM • Conflicts of interest • Euthanasia • Limitation of therapeutic effort
Course of illness	1	• Impact of the course of illness and prognosis of medical disorders in TDM • Considering comorbidities in TDM • Estimating patient prognosis • Using prognostic scores
Personal cognitions and biases	2	• Cognitions and heuristics in decision-making • Impact of personal emotions and preferences in decision-making • Unconscious bias • Strategies to minimize the negative impact of emotions and unconscious bias in TDM
Patients' perspectives	1	• Impact of patient preferences in TDM • Motivational interviewing strategies • Barriers and strategies to integrate patient's preferences in TDM
Patient´s family and social context	1	• The impact of the patient's context in TDM • Strategies to incorporate the patient's family and socioeconomic contexts in TDM
Patient-provider relationship	1	• Impact of patient-provider relationship in TDM • Types of patient-provider relationships and their impact on TDM • Shared decision making • Strategies to stablish different types of relationship according to the clinical context

Educational strategies of the course include self-instruction modules, application assignments, faculty symposia, and application seminars. Self-instruction modules used problem-based learning [17] to allow students identify their learning needs through clinical scenarios. Modules feature clinical vignettes to challenge students’ knowledge, present content, and apply lessons to case resolution. After completing each self-instruction module, students are invited to reflect on its contents and apply them to patients seen during their clinical rotations. These application assignments are recorded as videos and uploaded to the course´s portal.

Four 60-min faculty symposia feature guest specialists from various fields (e.g., digestive surgery, emergency medicine, geriatrics, palliative medicine, psychiatry) who share personal insights and patient stories related to the course’s topics. Students have opportunities to engage, discuss, and ask questions. Following each symposium, students participate in 90-min small-group application seminars, consisting of 10–13 students led by faculty from diverse specialties (e.g., endocrinology, family medicine, oncology, orthopedic surgery, among others). Using problem-based learning, these sessions encourage students to apply their learning to clinical cases, while faculty share their own practical experiences with TDM.

## Evaluation methods

### Study design

We conducted an observational, mixed-methods evaluation with three student cohorts (2020–2022). Quantitative data consisted of self-reports where students assessed the importance of various TDM domains and their ability to integrate these domains into therapeutic decisions with patients. Qualitative data included responses to open-ended questions evaluating students’ satisfaction with the course and its learning strategies. All procedures received approval from the PUC´s Institutional Review Board (IRB).

### Evaluation procedures

At the end of the course, all students completed an electronic before-and-after self-evaluation, assessing the importance of various domains in therapy decision-making and their self-efficacy in integrating these domains into clinical encounters. While ungraded, this evaluation was part of their end-of-course self-assessment, allowing students to reflect on their learning progress. Additionally, students from the 2020 and 2021 cohorts were invited to participate in follow-up assessments −2 years and 1 year after course completion, respectively- using a similar electronic tool. Former students were contacted through an email invitation from the course instructor and text messages sent by a teaching assistant from their cohort. The medical school provided demographic data for each cohort.

### Instruments

We developed the Therapy Decision-Making Domain Importance (TDMDI) and Therapy Decision-Making Domain Self-efficacy (TDMDS) Questionnaires to assess the importance of various domains in therapy decision-making and students’ self-efficacy in integrating these domains into patient therapy decisions (Supplements 2 and 3). Both instruments contain 10 parallel items rated on 5-point Likert scales, from ‘not important at all’ to ‘very important” for TDMDI, and ‘not competent at all’ to ‘very competent’ for TDMDS. Assessed domains include healthcare legislation, patient comorbidities, prognosis estimation, clinical evidence for treatments, treatment costs, patient preferences, family and social contexts, as well as personal biases and conflicts of interest in therapeutic decisions. Supplement 4 provides scale validation details. Additionally, we included quantitative assessments of learning strategies (from ‘very dissatisfied’ to ‘very satisfied’) and open-ended questions assessing student perceptions of the course and the learning strategies.

### Data analysis

#### Quantitative data analysis

Student demographics and responses were summarized using descriptive statistics, including means, standard deviations, and frequencies. Items in each scale were dichotomized: for the TDMDI, responses were categorized as ‘important’/ ‘very important’ vs other responses, and for the TDMSI as ‘competent’7 = / ‘very competent’ vs other responses. With 10 domains, scores range from 1 to 10.

To evaluate the course’s impact, the percentage of students rating domains as important and themselves as competent were compared pre-and post-course using McNemar tests. The average number of important or self-efficacious domains were compared using paired t-tests. Similar analyses were performed for the 1- and 2-year follow-up assessments to examine long-term effects. Chi-square and independent sample t-tests were used to compare follow-up results between the 2020 and 2021 cohorts, with no covariate adjustments P-values below 0.05 were considered statistically significant.

#### Qualitative data analysis

Open ended questions were coded using an inductive-deductive process following Content Analysis procedures [18]. One analyst initially coded the data, identifying emerging categories and sub-categories for each question. A second researcher reviewed the coding, and any discrepancies were discussed to reach full consensus. For external validation, additional researchers reviewed the coding process. Data were organized by the frequency of each category, and representative quotes were translated.

#### Mixed-methods integration

Quantitative and qualitative data were integrated using multiple strategies [19]. First, data were connected as the same participants provided both qualitative and quantitative information. A joint display, which represents quantitative and qualitative data simultaneously, was used to summarize perceptions towards learning strategies. Finally, at the reporting level, findings are presented in a continuous narrative, with mixed methods findings reported separately but within the same manuscript.

## Results

### Participants

A total of 367 students completed the course from 2020 and 2022. Approximately half identified as female (*n* = 183, 49.9%), with an average age of 22.2 ± 1.1 years. Most students were from the Santiago Metropolitan Area (75.5%, *n* = 277). The average Grade Point Average (GPA) was 6.2 ± 0.2 (on a 7.0), with only twenty students (5.4%) having previously failed a course. No statistically significant differences were observed between cohorts. All students completed the before- and after-course evaluation, and 112 and 97 students completed follow-up evaluations one year (88.2%) and two years (80.2%) after course completion.

### Satisfaction with learning strategies

**Fig. 1:**
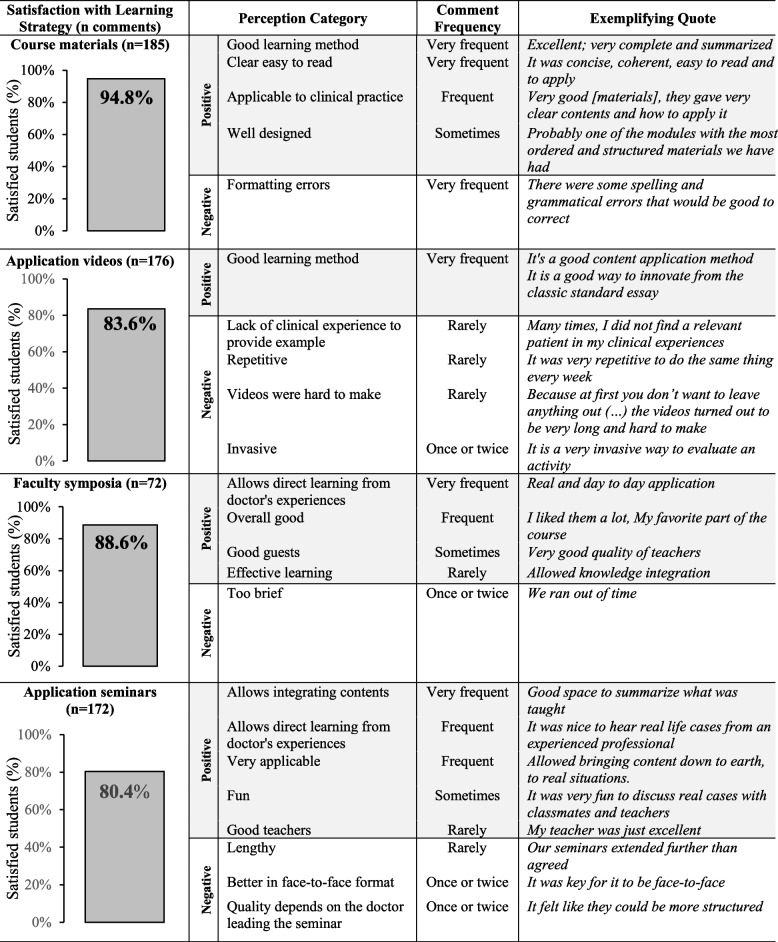
Joint display presenting quantitative and qualitative student perceptions with learning strategies

Students expressed high satisfaction with all learning strategies used in the course (Fig. 1). They appreciated the completeness, design, and readability of the materials though some noted formatting errors. Mandatory application assignments were considered effective for learning, although some students found them challenging when hadn’t yet encountered clinical scenarios to apply course concepts. Small-group seminars were valued for helping integrate theoretical contents and offering firsthand insights from practicing physicians. Faculty symposia provided students with opportunities to engage with faculty members, fostering deeper connection to practical applications.

### Course usefulness

By the end of the course, students appreciated the deeper understanding it provided of the various factors influencing therapy decisions. As one student noted: ‘*It helped me become aware of the different aspects that impact the decision-making process*’, This allowed to understand and integrate contents as ‘*the course´s contents wouldn't make sense to be taught in other clinical courses*’. At follow-up, students valued the comprehensive understanding of medical practice taught during the course. Students also considered the course learning helpful when providing patient care, highlighting that healthcare legislation and therapy´s evidence appraisal tools have been particularly useful.

### Important domains for therapy decision-making

**Fig. 2:**
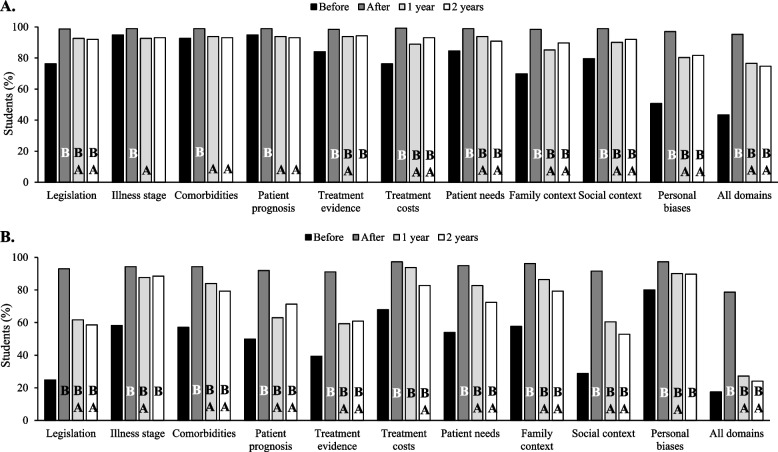
**A** Rate of students perceiving high importance of the different domains of the TDMDI in therapy decisions, **B** Rate of students perceiving high self-efficacy to include the different domains of the TDMDS in therapy decisions. Note: B = *p* < 0.05 compared to before the course; A = *p* < 0.05 compared to after the course

Before the course, students generally considered most domains assessed by the TDMDI to be important, with an average of 8.0 ± 2.4 domains rated as such per student. However, only 43% of students considered all 10 domains important for TDM (Fig. 2A). Following the course, the perceived importance of each domain increased (*p* < 0.001 for changes across all domains), and students considered an average of 9.9 ± 1.0 domains important (*p* < 0.001). A total of 95.2% of students considered all domains as essential for therapeutic decisions (*p* < 0.001). At follow up, the proportion of students assigning high importance to each domain had declined for most domains but remained higher than pre-course levels. On average, students rated 9.1 ± 2.3 domains as important for TDM at follow-up. Changes in perception at follow-up were similar between both student cohorts.

### Self-efficacy to include therapy decision-making domains in clinical practice

Figure 2B illustrates students´ self-assessed efficacy in considering various domains in TDM. Before the course, students reported feeling competent in an average of 5.2 ± 3.2 domains, with only 65 students (17.5%) rating themselves as capable or very capable of integrating all domains from the TDMDS into their clinical decisions. After the course, students’ self-efficacy significantly improved, with an average of 9.4 ± 1.5 domains reported as areas where they felt capable (*p* < 0.001). At follow-up, self-efficacy declined for most domains, with students rating themselves as competent in an average of 7.5 ± 2.5 domains. Although these changes represent a significant reduction from post-course assessment (*p* < 0.001 for most changes), it still marks a substantial improvement from baseline (*p* < 0.001 for all domains). No statistically significant differences were observed between student cohorts.

## Discussion

Teaching clinical reasoning is a vital component of medical education [20, 21]. This manuscript presents a structured curriculum to teach therapy decision-making (TDM), a skill often omitted from systematic instruction in medical schools and commonly left to clinical teachers to model during practice [9]. After implementing this TDM course with three student cohorts, we report high student satisfaction with the course´s educational strategies, high perceived importance for various domains relevant to TDM, [2] and a significant increase in self-efficacy for applying use these domains in clinical practice. Although one and two years post-course, students continued to value these domain’s importance, their self-efficacy in integrating them into practice decreased over time.

While many studies explore how clinicians make therapeutic decisions, [22] this curriculum uniquely provides a comprehensive educational approach to train medical students in the complexities TDM. Most educational research for medical and pharmacy students focuses primarily on appropriate prescription practices; [11, 13, 14, 23–27] however, while proper prescribing is critical, patient care often requires non-pharmacological intervention -including nutritional and behavioral counseling, surgery, both invasive and not-invasive procedures, among other therapeutic alternatives. Therapeutic reasoning has been identified as a crucial yet commonly component in medical education [9]. To address this gap, comprehensive TDM courses like the one detailed in this manuscript, should be systematically designed and integrated in medical curricula. Increased clinical exposure is insufficient, as studies show no clear correlation between clinic hours and reduced prescribing errors [28]. Therefore, targeted courses are essential to fill this educational need.

This course introduces several key innovations. First, it is strategically embedded in a clinically intensive semester, employing a context-learning approach that integrates practical and theoretical learning simultaneously rather than sequentially, where learning and application separate [16]. This approach enhances effectiveness, as context-learning helps students retain knowledge in a way that facilitates recall [16]. Additionally, the course incorporates self-learning modules and problem-based learning seminars, shown to enhance TDM skills more effectively than traditional methods [29]. Self-instruction modules guide students in learning new content, which they apply in individual assignments and small group seminars. Online platforms to support group work are another effective tool for TDM learning [30].

The course also features seminars and symposia with experienced physicians, a recommended strategy for enhancing therapeutic reasoning by covering both pharmacological and interventional therapies [6]. Students highly valued interactions and discussions with physicians from various specialties who shared their clinical reasoning in practice and in simulated. Finally, the course covered a comprehensive range of TDM topics, [1] including all available therapy options, which students continued to appreciate up to two years after completion. Future TDM educational programs should focus on comprehensive therapeutic reasoning that considers the full range of treatment options, including non-pharmacologic interventions. Such programs should emphasize critical domains in therapeutic decision-making, encourage reflection, and facilitate interactions with experienced providers around real and simulated clinical cases.

Baseline findings showed that students were aware of multiple factors relevant to therapeutic decisions but felt less confident about integrating them into clinical practice. This pattern is typical for medical students beginning their clerkships and building clinical skills [31]. Notably, students initially placed greater importance on ‘traditional medical elements’ for therapeutic decisions (e.g., disease stage, patient prognosis, comorbidities) over ‘non-traditional domains’ (e.g., personal biases, healthcare legislation, treatment costs), likely reflecting a biomedical focus within the school’s curriculum. After the course, while students’ self-perceptions and confidence in integrating different TDM elements increased, these perceived importance and self-confidence levels declined for many domains over time, returning to pre-course levels. Since teaching on these non-traditional TDM aspects is rare, faculty might not emphasize them during precepting, leading students to view them as less critical as they progress training. These results underscore the need for formal TDM instruction and a longitudinal integration within the curriculum [32]. Training on these domains should be integrated across all clerkships and rotations, emphasizing their relevance to medical care. Additionally, training faculty to recognize and value non-traditional domains in TDM is essential. Brief booster workshops during later semesters could help sustain self-efficacy throughout student training, as even short (1–2-h) sessions have been shown to enhance prescription skills among new providers [33].

While this project demonstrates notable strengths, it also has limitations that warrant discussion. Firstly, the curriculum was developed based on the clinical experience of DGH and LML, whose combined expertise exceeds 40 years, as well as existing literature on clinical reasoning. Future studies could strengthen the curriculum’s validity by employing a consensus-based approach. Secondly, the course has seen variable implementation since 2020. Initially, Coronavirus Disease 19 restrictions necessitated virtual formats for certain instructional strategies (e.g., integration seminars), limiting interactions between students and physicians. Additionally, clinical exposure was restricted for the 2020 and 2021 cohorts, leading to variations in patient interactions and opportunities for practical application. Furthermore, as is typical for academic programs, course adjustments have been made annually to improve student learning. Despite these modifications, no significant outcome differences were noted between cohorts.

A third limitation involves the completion rate of follow-up assessments: while all students completed pre- and post-course evaluations follow-up assessments were completed by 88.2% of students after one year and 80.2% after two years. Institutional Review Board restrictions prevented collection of identifying information (e.g., gender, age), limiting the analysis of selection bias. Nonetheless, our sample size remains robust enough to detect at least a 15% difference in perceived importance and self-efficacy between cohorts, which is a meaningful difference from an educational standpoint.

Moreover, the study relied on the TDMDI and the TDMDS to measure students’ perceptions, which are low level learning outcomes in educational evaluations [34]. Although these measures have high psychometric properties that ensure data reliability, future assessments should also target higher level of learning outcomes, such as behavioral changes and clinical performance. Prior research suggests that self-confidence in prescribing skills often does not correlate with objective competency, [15] though it is a common evaluation method in prescription education [10, 11]. Future evaluations could incorporate Objective Structured Clinical Examinations (OSCEs) to measure the impact of the course on more relevant student learning outcomes.

Finally, as the course developers and instructors also conducted the evaluations, a potential conflict of interests exists, which could influence data interpretation. To address this, we have provided comprehensive details on study procedures, included measure validation data in the supplementary materials, and reported all findings transparently. These procedures help ensure credibility of our results.

## Conclusions

We present an innovative educational course on therapeutic decision making (TDM) for medical students. Participation in the course increased students’ perceived importance of key domains in therapeutic decisions and boosted their self-efficacy in applying these domains in clinical care. Students highly valued the course’s diverse educational strategies, such as self-instruction modules, practical assignments, application seminars, and faculty-led symposia, underscoring the benefit of using multiple teaching methods to enhance learning. Future TDM training should incorporate courses that encourage reflection on the wide range of therapeutic options available in medicine, cover essential domains for TDM, and foster constructive interactions between students and experienced clinicians.

## Supplementary Information

Supplementary Material 1

## Data Availability

The data that support the findings of this study are available from the corresponding author, DGH, upon reasonable request.
