# Supplementary material for: Teaching therapy decision-making to medical students: a prospective mixed-methods evaluation of a curricular innovation
Source: BMC Med Educ. 2024 Dec 26;24:1533. doi: 10.1186/s12909-024-06421-y (PMC11670388; doi:10.1186/s12909-024-06421-y)
Supplement: Supplementary file 1 — Supplementary Material 1 [file 12909_2024_6421_MOESM1_ESM.docx]

Supplement 1. Description of the Pontificia Universidad Católica School of Medicine

In Chile´s educational system, students can initiate medical education after high school. The Pontificia Universidad Católica de Chile´s Medical School is known for its innovative approach towards medical education, ranking second in Latin America [32]. Currently, it has about 750 students and 600 faculty providing learning experiences in 4 hospitals and 6 ambulatory medical centers. In 2015, the Medical School changed its curriculum from seven to six years with the goal of integrating student learning and facilitating transition towards medical residencies [33]. The career is split in three phases: preclinical (4 semesters) where students learn about the foundations of medicine (morphology, physiology, pharmacology, etc.), clinical (7 semesters) where students learn about clinical practice shadowing physicians in their practice, and internships (3 semesters) where students get significant supervised clinical experiences across the most common medical specialties. As part of this curriculum innovation, all students provided informed consent to participate in all medical education evaluation activities.

The Therapy Decision Making course is part of a 4-sequential course of clinical integrative courses: Diagnostic Reasoning, Therapy Decision Making, Complex Decisions in Medical Care, and Health Promotion and Disease Prevention across the Lifespan. The goal of these courses is to integrate contents of ethics, legal medicine, evidence-based medicine, health economics, public health, social determinants of health, medical psychology, and clinical medicine in a continuum during the clinical formation of students.

**References:**

1. QS World University Rankings. 2023. Rankings by Subject 2023: Medicine. [accessed 2024 Aug 20]. https://www.topuniversities.com/university-subject-rankings/medicine/2023?region=Latin%20America
2. Cisternas M, Rivera S, Sirhan M, Thone N, Valdés C, Pertuzé J, Puschel K. 2016. Curriculum reform at the Pontificia Universidad Católica de Chile School of Medicine. Revista Médica de Chile. 144(1):102–107. doi:10.4067/s0034-98872016000100013.

Supplement 2. Therapy Decision-Making Domain Importance Scale (TDMDI)

Please complete the following scale rating how important you think that each item is for therapy decisions. First, respond thinking at the beginning of the semester, and then, now that you have completed the course

|  |  | At the beginning of the semester | | | | |  | Now | | | | |
| --- | --- | --- | --- | --- | --- | --- | --- | --- | --- | --- | --- | --- |
|  |  | Not important at all | Little important | Neutral | Important | Very important |  | Not important at all | Little important | Neutral | Important | Very important |
| Laws are important for therapy decisions |  |  |  |  |  |  |  |  |  |  |  |  |
| Patients’ illness stage is important for therapy decisions |  |  |  |  |  |  |  |  |  |  |  |  |
| Patients’ comorbidities are important for therapy decisions |  |  |  |  |  |  |  |  |  |  |  |  |
| Patients’ prognosis is important for therapy decisions |  |  |  |  |  |  |  |  |  |  |  |  |
| Treatment evidence is important for therapy decisions |  |  |  |  |  |  |  |  |  |  |  |  |
| Treatment costs are important for therapy decisions |  |  |  |  |  |  |  |  |  |  |  |  |
| Patient needs and preferences are important for therapy decisions |  |  |  |  |  |  |  |  |  |  |  |  |
| Patients’ family context is important for therapy decisions |  |  |  |  |  |  |  |  |  |  |  |  |
| Patients’ social context important for therapy decisions |  |  |  |  |  |  |  |  |  |  |  |  |
| Personal biases and conflicts of interest are important for therapy decisions |  |  |  |  |  |  |  |  |  |  |  |  |

Supplement 3. Therapy Decision-Making Domain Self-efficacy Scale (TDMDS)

Please complete the following scale rating how competent you feel including each item in therapy decisions. First, respond thinking at the beginning of the semester, and then, now that you have completed the course

|  |  | At the beginning of the semester | | | | |  | Now | | | | |
| --- | --- | --- | --- | --- | --- | --- | --- | --- | --- | --- | --- | --- |
|  |  | Not competent at all | Little competent | Neutral | Somewhat competent | Very competent |  | Not competent at all | Little competent | Neutral | Somewhat competent | Very competent |
| I can include laws in therapy decisions |  |  |  |  |  |  |  |  |  |  |  |  |
| I can include patient’s illness stage in therapy decisions |  |  |  |  |  |  |  |  |  |  |  |  |
| I can include patient’s comorbidities in therapy decisions |  |  |  |  |  |  |  |  |  |  |  |  |
| I can include patient’s prognosis in therapy decisions |  |  |  |  |  |  |  |  |  |  |  |  |
| I can include the treatment evidence in therapy decisions |  |  |  |  |  |  |  |  |  |  |  |  |
| I can include the treatment costs in therapy decisions |  |  |  |  |  |  |  |  |  |  |  |  |
| I can include the patient’s needs and preferences in therapy decisions |  |  |  |  |  |  |  |  |  |  |  |  |
| I can include the patient’s family context in therapy decisions |  |  |  |  |  |  |  |  |  |  |  |  |
| I can include the patient’s social context in therapy decisions |  |  |  |  |  |  |  |  |  |  |  |  |
| I can include my personal biases and conflicts of interest in therapy decisions |  |  |  |  |  |  |  |  |  |  |  |  |

Supplement 4. TDMDI and TDMDS validation

**Test re-test reliability**

In 2020, 119 students completed the TDMDI and TDMDS questionnaires at the beginning of the course, and then as part of a before-and-after assessment at the end of the course. Pearson correlations between item responses between baseline evaluation and the before component of the end of the semester assessment were 0.912 and 0.892 for the TDMDI and TDMDS, respectively.

**Internal reliability**

Internal scale consistency was assessed using Cronbach´s alpha for before and after components separately. Both analyses had a total sample of 367 students. Cronbach´s alpha for each scale at each time point are presented in the following table:

| **Instrument** | **Baseline**  **Cronbach´s alpha** | **Before course component of post-course assessment**  **Cronbach´s alpha** |
| --- | --- | --- |
| Therapy Decision-Making Domain Importance Scale | 0.9132 | 0.9592 |
| Therapy Decision-Making Domain Self-efficacy Scale | 0.9225 | 0.9184 |
